# Inferring Cultural Landscapes with the Inverse Ising Model

**DOI:** 10.3390/e25020264

**Published:** 2023-01-31

**Authors:** Victor Møller Poulsen, Simon DeDeo

**Affiliations:** 1 Department of Social and Decision Sciences, Carnegie Mellon University, 5000 Forbes Avenue, Pittsburgh, PA 15213, USA; 2 Santa Fe Institute, 1399 Hyde Park Road, Santa Fe, NM 87501, USA

**Keywords:** machine learning, history, archaeology, anthropology, religion, cultural evolution, inverse Ising model, spin glass, robust statistics

## Abstract

The space of possible human cultures is vast, but some cultural configurations are more consistent with cognitive and social constraints than others. This leads to a “landscape” of possibilities that our species has explored over millennia of cultural evolution. However, what does this fitness landscape, which constrains and guides cultural evolution, look like? The machine-learning algorithms that can answer these questions are typically developed for large-scale datasets. Applications to the sparse, inconsistent, and incomplete data found in the historical record have received less attention, and standard recommendations can lead to bias against marginalized, under-studied, or minority cultures. We show how to adapt the minimum probability flow algorithm and the Inverse Ising model, a physics-inspired workhorse of machine learning, to the challenge. A series of natural extensions—including dynamical estimation of missing data, and cross-validation with regularization—enables reliable reconstruction of the underlying constraints. We demonstrate our methods on a curated subset of the Database of Religious History: records from 407 religious groups throughout human history, ranging from the Bronze Age to the present day. This reveals a complex, rugged, landscape, with both sharp, well-defined peaks where state-endorsed religions tend to concentrate, and diffuse cultural floodplains where evangelical religions, non-state spiritual practices, and mystery religions can be found.

## 1. Introduction

If we want to understand the powers and potentials of the human species—the landscape of both what has been, and could be, done—we are driven to make comparisons across vast ranges of time and culture. In these cases, data is not only missing, but differentially missing [1]. To analyze, at the same time, a contemporary culture of the digital age, and one that vanished five thousand years ago, requires careful accounting. There is both the intellectual challenge of making best use of what information reaches us, and an ethical imperative to treat long-lost cultures—and marginalized, under-studied, or minority cultures that survive today—on an equal epistemic footing with the dominant, often “WEIRD” [2] ones, for whom data is both more abundant and more complete.

Appropriate modeling of small, and potentially biased, data is a challenge. Replacing missing values with “no” or “not present”, for example, is the fallacy of taking absence of evidence for evidence of absence. Replacing them with the median answer, or the best match, from the remainder of the data makes unfamiliar cultures clones of the ones we know. Replacing them with “a fifty-fifty mixture of present and absent” is not much better: it attributes the lack of knowledge in the observer to a lack of coherence in the original culture; because we do not know what they did, we assume they did not, either. All these challenges are exacerbated in the “small data” limit common in studies of cultural evolution—archives with hundreds of data points, rather than the millions on which machine-learning algorithms are usually trained and tested.

This paper addresses the challenge of inferring cultural landscapes in deep time [3,4]. We show how to extend a commonly used workhorse of machine learning—the Inverse Ising Problem with minimum probability flow [5]—to the kind of sparse, under-sampled, and potentially biased samples of the historical record. While standard approaches can give misleading answers, we show how a set of carefully constructed modifications and extensions can provide new ways to ask basic questions about the evolution of human culture. We then demonstrate the power, and potential, of cultural landscape construction with an analysis of a curated subset of the Database of Religious History (DRH) [4,6].

## 2. Methods

The goal of our analysis is the construction of a cultural landscape: a general model of what makes different cultural patterns more or less likely to appear in the course of time. To be more specific, imagine that we have a set of “characteristics”—aspects of a culture that we care about, and which can be represented with a binary answer such as YES or NO, TRUE or FALSE, PRESENT or ABSENT, and so on. A particular setting of all the answers is called a configuration, and a landscape model says, for any particular configuration, how likely it is to appear.

Depending on how the experts understand the questions, the landscape derived from it might characterize, on one extreme, the patterns of behavior that could emerge in an individual—or, on the other extreme, the kinds of patterns that entire societies might explore across the span of human history. In the case treated here, we have cross-cultural data on religious groups in different cultures and time periods from 10,000 BCE to the present day; one group characteristic we consider, for example, is “Are supernatural beings believed to mete out punishment?” while another is “does membership in this group require participation in small-scale (private, household) rituals?” and a third is “does membership in this religious group require sacrifice of children?”

A landscape model, could it be found, would be a powerful tool for systematic investigation of how societies compose these different characteristics together to form the foundation of a stable cultural practice. We might want to know, for example, whether a “yes” answer to a belief in punishing gods makes it more likely for the religion to rely on small-scale rituals, all other things being equal, and how this relationship might be mediated by the presence of extreme practices such as child sacrifice.

Being able to answer these questions would provide important empirical constraints to more fundamental models. One model, for example, might understand child sacrifice as an extreme example of costly signals of devotion in a social context, otherwise disconnected from the metaphysical account the religion provides about god, while another might see the practice as something that could only be conceivable against a particular conceptualization of the relationship between humankind, nature, and the transcendent (see, e.g., Ref. [7] for discussion). The two models will make different predictions of how the practice co-varies with other characteristics.

Answers to questions such as these cannot be simply read off from the data, however, because religions with and without a belief in supernatural punishment generically differ on a wide range of characteristics, all of which might impact a violent practice such as child sacrifice. The correct answer requires a comparison to a fiducial culture that differs in only one characteristic. The space of configurations expands exponentially, and probing fundamental questions requires knowledge not just of the religions we happen to have observed, but the larger, law-governed landscape of what combinations—including those never observed in human history—are more or less likely.

A landscape model allows us to investigate which features of a religion most strongly couple with others. It provides insight into how different aspects of a religion bundle together [8], with a small number of distinct patterns of yes/no answers, as might happen if religions were divided into (for example) Axial and pre-Axial types. It would even allow to us identify practices that have yet to emerge—unexplored regions of cultural-evolutionary space. A more prosaic, though no less important, use of a landscape model is to predict missing data. For a long-lost culture, for example, whose metaphysical beliefs are unknown, a landscape model can predict the probabilities of different combinations of epistemic commitments on the basis of its material culture.

### 2.1. From Physics to Machine Learning: An Introduction to the Inverse Ising Problem

Inferring such a fitness landscape from data requires us first to specify the structure of the landscape itself—the spectrum of ways in which it allows one aspect of a pattern to make other aspects more or less likely. In traditional approaches, such as logistic regression, one chooses, ahead of time, a small number of possible effects, based on an explicit model; with a hundred data points, for example, one might try to learn—estimate—three or four regression coefficients.

When learning a landscape, by contrast, the number of parameters is very large—often comparable to, or even very much larger than, the number of observations [9]. The particular model we consider in this paper is a very general form of a neural network known in the machine-learning literature as the “unrestricted Boltzman machine”, and (in the physics literature) as the “inverse Ising problem” [10].

The inverse Ising model has been applied, with great success, to data ranging from neuroscience [11,12], the immune system [13], and the fitness landscapes of HIV [14], to animal behavior [15,16], political polarization and voting behavior [17,18], and linguistics [19]. It has also been used as a general model of generic complex cultural practices in cultural evolution [20]. In one common notation choice, the inverse Ising model says that the probability of observing a configuration *i* is

(1) $$p_{i}=\frac{\exp E_{i}\left(\vec{\theta }\right)}{Z(\vec{\theta })},$$

where $\vec{\theta }$ are the parameters (to be estimated); $Z(\vec{\theta })$, traditionally called the “partition function”, is the normalization constant; and the “energy”, $E_{i}\left(\vec{\theta }\right)$, of a particular configuration is given by

(2) $$E_{i}\left(\vec{\theta }\right)=\sum_{a,b;a>b}J_{ab}\sigma_{a}\sigma_{b}+\sum_{a}h_{a}\sigma_{a},$$

where $\sigma_{a}$ is the truth value of the *a*th entry in configuration *i*; by convention, YES is +1, and NO is −1; there are n(n−1)/2 of the “*J*” parameters (the “pairwise couplings”), and *n* of the “*h*” parameters (the “local fields”).

In general, physicists take the *J* and *h* values (or the probability distributions they are drawn from) as given, and try to understand the properties of the resulting distribution [21]. The converse problem, which we consider here, is to infer the “best fit” *J* and *h* that can predict the observed frequencies of the occurrence of different configurations in a dataset.

As first noted by E.T. Jaynes [22], the form of Equation (2) means that, properly estimated, *p* is the distribution with maximum entropy that, at the same time, matches the observed means and pairwise correlations; i.e., those found by averaging over all the observed vectors, ${\vec{\sigma }}_{d}$, in the dataset D,

(3) $$\sum_{i}\sigma_{a}p\left(i\right)=\frac{1}{\left|D\right|}\sum_{d\in D}\sigma_{a,d}\;\mathrm{and}\;\sum_{i}\sigma_{a}\sigma_{b}p\left(i\right)=\frac{1}{\left|D\right|}\sum_{d\in D}\sigma_{a,d}\sigma_{b,d}$$

Such models embody a kind of inverted form of Occam’s Razor: make the model just sophisticated enough to explain only the least complicated features of the data at hand, leaving everything else maximally undetermined. Surprisingly enough, this works: as has been repeatedly discovered, higher order correlations often “come along for the ride”, emerging spontaneously when the pairwise constraints of Equation (3) are satisfied [12,23,24]. Despite its simplicity, Equation (2) can capture a great deal of the real variability in complex systems, and many of the most celebrated successes of machine learning are, at heart, adaptations of this insight [25].

### 2.2. Minimum Probability Flow

Finding the values of *J* and *h* that satisfy Equation (3) is exponentially hard, because it requires averaging over all $2^{n}$ configurations in the probability distribution, Equation (1). We can rephrase the problem, however, as trying to find the Ising-model distribution, $p_{i}\left(J_{ab},h_{a}\right)$, that best fits the true (or “data”) distribution, $p_{i}$, where the “best fit” is the one that minimizes the Kullback–Leibler divergence,

(4) $$K\left(\vec{\theta }\right)=\sum_{i\in C}p_{i}\log_{2}\frac{p_{i}}{p_{i}\left(\vec{\theta }\right)},$$

where C is the (exponentially large) set of all $2^{n}$ configurations. The $\vec{\theta }$ that minimizes Equation (4) produces a $p_{i}\left(\vec{\theta }\right)$ which is minimally distinguishable, in a basic information-theoretic sense, from the true distribution $p_{i}$.

Minimizing Equation (4) directly, however, still requires multiple sums over C. The insight of MPF [5] is that, given a collection of observed configurations, D, Equation (4) can be approximated by minimizing the “probability flow”. When a parameter choice $\vec{\theta }$ is a poor match to the data, probability tends to flow “away” from data states to non-data states. Up to constant factors, we can approximate Equation (4) as

(5) $$K\left(\theta \right)=\sum_{j\in D}\left(\sum_{i\in N\notin D}\Gamma_{ij}\left(\vec{\theta }\right)\right),$$

where $\Gamma_{ij}\left(\vec{\theta }\right)$ is the rate of flow from state *j* to state *i* for parameter choice $\left(\vec{\theta }\right)$, and N is a set of “neighbouring” non-data configurations. Minimizing Equation (5) is a tractable task; in contrast to Equation (4), the sums are no longer over C, but a radically smaller set of observed data, D, and a well-chosen N. MPF is related to a basic method in machine learning, contrastive divergence [26], with the principle advantage, for our purposes, of having a well-defined, epistemically principled, objective function.

### 2.3. Improvements and Extensions to the MPF Algorithm

In this section, we present a series of improvements and extensions to the basic MPF algorithm. These include both apparently minor, but critical, variations in the basic algorithm, and a new extension and derivation. We are particularly grateful to the authors of ConIII [27], whose implementation, and clear discussion, of MPF enabled us to debug and test our own code.

Section 2.3.1 and Section 2.3.2 present a pair of improvements to the basic algorithm; these provide significant boosts in performance and accuracy on sparse social and cultural data. Section 2.3.3 shows how to handle inconsistencies between different observers (or inconsistencies within the same observer), and Section 2.3.4 shows how the same tools also allow us to account for uneven sampling in time or space. Finally, Section 2.3.5 describes a novel extension to the MPF algorithm, Partial-MPF, which enables us to handle missing data in a principled fashion.

#### 2.3.1. Nearest-Neighbour Sampling

In the original version of the MPF algorithm, flow is computed from the observed configurations (“data states”) to a subset of other configurations, explicitly excluding flow into any other data states. It is equally valid, under the MPF approximation, to allow flow into states that do appear elsewhere in the data; this can be seen at line A-6 of Ref. [5], where you can interchange the order of the derivative and the summation. This alternative choice is the default under ConIII.

Our experiments find that the alternative choice provides greatly improved out-of-sample performance, because the exclusion biases the algorithm against configurations near a metastable peak. With this change in hand, the function to be minimized is

(6) $$K\left(\theta \right)=\sum_{j\in D}\left(\sum_{i\in N(j)}\Gamma_{ij}\left(\vec{\theta }\right)\right),$$

A natural choice is to set N(j) to include states within a certain Hamming distance of *j*; the original MPF paper considered states that differed from the data state at one position, i.e., $N_{1}\left(j\right)$; we also consider a strategy which uses states up to two ($N_{2}\left(j\right)$) Hamming units away. Since |N(j)| is the same for all *j*, this provides equal weighting to all data states. (It is also possible to consider randomly chosen neighbours; however, this tends to give significantly decreased performance; the MPF algorithm performs best when it is allowed to focus on reasonably nearby variations from the observations.)

#### 2.3.2. Regularization Constraint

Minimizing Equation (6) is equivalent to (attempting to) maximize the posterior log-probability of the data given the model. A proper Bayesian analysis, however, should include not just the posterior, but a prior over the parameters themselves,

(7) $$K^{\prime }\left(\theta \right)=K\left(\theta \right)-\lambda |D||N|\log P(\vec{\theta }),$$

where λ is a constant, and $P(\vec{\theta })$ is the probability of a particular choice for *J* and *h*.

It is natural to choose $P(\vec{\theta })$ so that, all other things being equal, smaller values are preferred; this is sometimes known as a regularization penalty, which often provides significant benefits to out-of-sample prediction [28]. Without regularization, models tend to overfit, producing unreasonably low probabilities for configurations that happen not to appear in the data.

If we assume that *J* and *h* are distributed as a Gaussian—what is sometimes known as the L2-norm—we have

(8) $$K^{\prime }\left(\theta \right)=K\left(\theta \right)-\lambda |D||N|\sum_{k=1}^{N_{p}}\frac{\theta_{k}^{2}}{2},$$

where the value of λ encodes the variance in the Gaussian; a larger λ corresponds to a smaller variance.

The optimal choice for λ depends on $P(\vec{\theta })$, which is, in general, unknown. It can be estimated, however, by cross-validation: if there are *m* datapoints, fit the data using m−k datapoints (the training set), and compute the log-likelihood for the remaining *k* datapoints (the test set). In this paper, we take *k* equal to one, i.e., leave-one-out cross-validation. Repeating this for all possible choices of the left-out observation, and then averaging the result, allows us to estimate the performance of the fit as a function of λ.

#### 2.3.3. Inconsistent Data

In some case—for example, in about 17% of religious groups in the DRH data used below—we have inconsistent coding, where multiple, incompatible answers exist for the same configuration. This can emerge when different observers interpret a question, or evidence, in different ways, or have different examples in mind. In the DRH, it most commonly appears when the same observer flags a feature as less straightforward than it appears; for example, “Iban traditional religion” is inconsistently coded for whether the religion had scriptures, with the coder citing it as a “borderline case” and answering both “yes”, and “no”. Another example is “Unitarian Universalism” (UU), where the same observer coded belief in afterlife as both “yes”, and “no”, noting that some UUs do, and some do not, believe in an afterlife. A proper accounting of the landscape ought to allow for both.

To make explicit use of inconsistent data requires an error model, and there are two natural choices. Consider, as an example, two observers who provide inconsistent answers, for the same system, to three binary questions: $j_{1}$ gives {1,1,0}, while $j_{2}$ gives {1,0,1}. If we assume that, for each observer, their best answer to one question is dependent upon all the others, we can include both records, with a weighting term, $w_{j}$, which captures the epistemic uncertainty

(9) $$K\left(\theta \right)=\sum_{j\in D}\left(\sum_{i\in N(j)}w_{j}\Gamma_{ij}\right),$$

where $w_{j_{1}}=w_{j_{2}}=1/2$. Alternatively, one can take inconsistencies as evidence of uncertainty question by question—the “independent” model. Then we interpret the observations $j_{1}$ and $j_{2}$ as indicating that observers are, in general, uncertain about the answers to questions two and three, with independent probabilities of “yes” for each 1/2. In this case, one includes not only the observed records ($r_{1}=\left\{1,1,0\right\}$, $r_{2}=\left\{1,0,1\right\}$) but also the unreported combinations $r_{3}=\left\{1,1,1\right\}$ and $r_{4}=\left\{1,0,0\right\}$, each with weight 1/4.

Both choices imply that differences between observers trace back, not to uncertainty about a fixed reality, but rather to fluidity in the practices themselves, where both answers are equally valid depending on the details of time and place. The examples presented above are the most common form of inconsistency, and this argues in favor of the independent model.

#### 2.3.4. Correcting for Non-Uniform Weighting across Time and Space

Cultural data is often unevenly sampled. We have more examples from the present than the distant past; more from high-GDP countries than from low-GDP countries; more from dominant cultures in a region than from marginalized or minority ones.

This can lead to bias in our landscape estimation. If we have, for example, 20 observations from cultures of Type A (the “contemporary developed world” sample), and only 10 observations from cultures of Type B (the “understudied”, or “minority”, sample), then a naive use of the data would tend to lead to landscapes that made Type-A cultures look more stable than Type-B cultures, and would produce accounts of the interlocking constraints that made Type-A cultures look more natural than Type-B cultures.

Often, however, we will know from archival records or field reports that groups exist, even if we know nothing about them, which allows us to estimate the sampling bias. With such an estimate in hand, Equation (9) allows us to re-weight observations to compensate.

#### 2.3.5. Partial-MPF: Accounting for Missing Data

Handling missing data is a challenge. Consider an observation such as the following,

(10) $$\begin{array}{c} j=\{1,0,X,X\}, \end{array}$$

where answers to the last two questions are not provided. The function that MPF minimizes, Equation (5), can only be calculated for fully specified data, and so a natural response is to perform data imputation: for example, replacing missing answers with the most common responses for that question in the remainder of the data.

While naive imputation methods are often suggested in machine-learning tutorials, they are, in the final analysis, an epistemic fallacy: they replace what is unknown by what is known, and assume that what has not been seen looks like what has. In qualitative work, such a fallacy would be obvious. An archaeologist would not suggest, for example, that the metaphysical beliefs of a long-vanished civilization should match the median beliefs of civilizations today.

A better way to solve this problem, which we refer to as “Partial-MPF”, is to dynamically infer the missing data from the best estimates of the parameters $\vec{\theta }$; i.e., to work not with a particular completion for *j*, but a distribution over, in this case, the four possible values, $j_{1}$, {1,0,0,0}, $j_{2}$, {1,0,0,1}, $j_{3}$, {1,0,1,0}, and $j_{4}$, {1,0,1,1}, found using Equation (1).

When the amount of missing data is small (in practice, fewer than 10 missing values per configuration, independent of the total number of questions), the distribution can be computed exactly. For an observation with *m* missing values, we expand the observation into the $2^{m}$ different combinations; compute the weights, $w_{j}\left(\vec{\theta }\right)$, for each combination; and combine them together as in Equation (9). This is somewhat like the “expectation-maximization” step suggested by Ref. [29] for missing data, but with probabilistic weightings that preserve continuity in the derivative.

Performing this correctly requires care, and there are three alterations we have to make to the basic algorithm. First, we must update the weights $w_{j}\left(\vec{\theta }\right)$ as we move through parameter space. Second, because the weights depend on $\vec{\theta }$, this changes the form of the derivative $dK\left(\theta \right)/d\vec{\theta }$. Third, when considering a configuration with missing data, we have to restrict its neighbour space to include only those configurations that differ in a known question.

Importantly, while inference of the missing data is exact, $K(\vec{\theta })$ is still only an approximation, and so minimizing $K(\vec{\theta })$ will be in slight tension with the new (exact) inference step that Partial-MPF takes. As we will see in Section 4.3, this is not a show stopper, and our treatment of missing data is, in practice, much more effective than standard alternatives.

## 3. Data

Our case study draws on data from the Database of Religious History (http://religiondatabase.org, accessed on 12 October 2022) (DRH) [4,6]. The DRH, an ongoing project based at the University of British Columbia, includes a peer-reviewed collection of information about religious groups in both the contemporary, historical, and archaeological record, in the form of coded answers to standardized question sets (“polls”, in the DRH) [4,30,31].

The DRH is organized hierarchically, such that some “super” questions (e.g., “Is a spirit-body distinction present?”) have sub-questions (e.g., “Is spirit-mind conceived of as having qualitatively different powers or properties than other body parts?”), and even sub-sub-questions. For this case study, we limit ourselves to super questions, since sub-questions are contingent on answers to super questions. This limits the number of questions from 1133 to 171. The majority of the questions are binary questions, and so are a natural fit to the Inverse Ising method. When we limit ourselves to questions that ask for binary answers, this further limits the number of questions from 171 to 149, and the number of records from 838 to 835.

The DRH is under continuous development. In this preliminary analysis, intended to demonstrate the methods and the basic ideas behind landscape construction, we focus on a subset of 20 questions, and do not correct for potentially uneven sampling of groups by time or place. We start by selecting the questions with the fewest unanswered questions across records, and then select all records (i.e., religious groups) that have five or fewer missing answers. Additionally, selecting only civilizations from the “group” poll [30], leaves us with a final data set of 407 civilizations. We infer parameters by running the Partial-MPF algorithm on these observations. See Appendix B Table A1 for the full list of questions, and Appendix B Table A3 and Table A4 for all religious groups in our curated dataset.

## 4. Results: Simulations

We first present the results of simulations; these confirm that our extensions to the basic MPF algorithm provide critically important improvements to the quality of the fit. To do this, we create large numbers of “imaginary” landscapes, where the underlying parameters have statistics similar to those observed in the real world. We take *n*, the number of YES/NO questions, equal to 20, and we draw the parameters $J_{ab}$ and $h_{a}$ from a Gaussian distribution. We then simulate data as draws from this underlying distribution, using the Metropolis–Hastings algorithm, altering it in different ways to take into account how real-world data is distorted by the data-gathering process.

With these simulated datasets in hand, we use our different extensions to the MPF algorithm to attempt to infer the underlying true parameters. We quantify the performance of our algorithms by direct calculation of the Kullback–Leibler divergence in the inferred distribution (corresponding to inferred parameters ${\hat{J}}_{ab}$ and ${\hat{h}}_{a}$) from the true distribution (which, in our simulations, is known—it is just the distribution produced by the original $J_{ab}$ and $h_{a}$),

(11) $$\mathrm{KL}=\sum_{i\in C}p_{i}\left(J_{ab},h_{a}\right)\log\frac{p_{i}\left(J_{ab},h_{a}\right)}{p_{i}\left({\hat{J}}_{ab},{\hat{h}}_{a}\right)}.$$

When KL is close to zero, the inferred distribution is hard to distinguish from the true distribution—i.e., it is a good fit. KL has a number of useful properties that allow it to play the role of “mean squared error” for probability distributions [32], quantifying the relative error in reconstruction and prioritizing accurate reconstruction of the more common states.

In general, reconstruction performance will depend upon the parameters of the distribution from which the test values $J_{ab}$ and $h_{a}$ are drawn. For our particular case of N=20, we choose this to be a Gaussian with mean zero, and σ ranging between 0.01 and 1.0.

When σ is small, the constraints are very weak and we are in a near-random or “dispersed” regime. As σ becomes larger, we enter what we call the “ordered” regime up to σ of approximately 0.25, where constraints are strong enough to produce peaks where data tends to cluster; practically speaking, this is where most real-world systems, including the DRH, tend to be found. For completeness, we consider yet larger σ values: going above 0.25 we enter the “near critical” regime, where peaks become sufficiently strong to produce large-scale order, and, finally, what we call the “critical” regime, above 0.5, where the distribution is near, or past, the spin-glass phase transition.

### 4.1. Regularization and Cross-Validation Greatly Improve Performance

**Figure 1:**
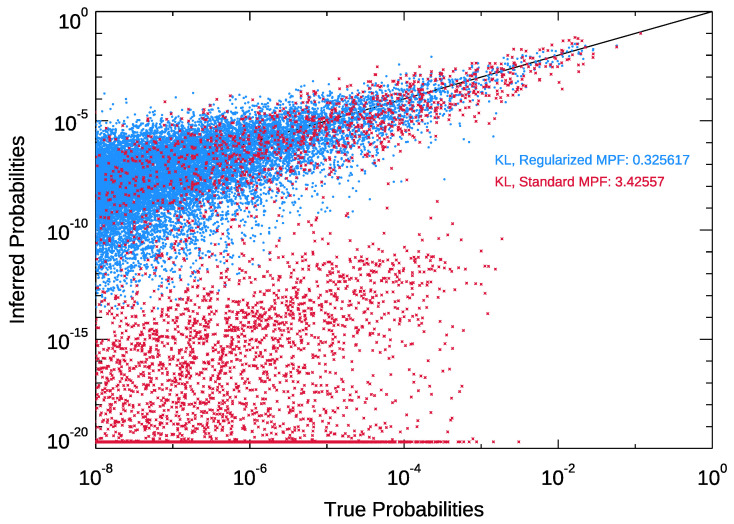
Regularization corrects for overfitting. A sample reconstruction of the $2^{20}$ (≈1 million) probabilities for a landscape, based on 256 datapoints. Without the regularization constraint (red points), the model underestimates the probabilities of some reasonably common configurations. The effect is largely controlled for when using regularization with cross-validation (blue points).

Regularization using the λ parameter significantly improves our ability to estimate the underlying landscape, making reliable extraction possible with very small amounts of data. An example is shown in Figure 1, where we take a particular simulated dataset (with σ equal to 0.5), and compare the probabilities estimated using the baseline MPF (i.e., without regularization), to our regularization method where λ is estimated using leave-one-out cross-validation.

The regularized model is not only better at estimating the probability of the peaks of the landscape (the more likely, high-probability configurations), it also avoids overfitting to less common configurations. Standard MPF, by contrast, can sometimes recover very large values for the $J_{ab}$ parameters, leading it to underestimate the vast majority of the less likely configurations (*p* less than $10^{-2}$). For Standard MPF, sometimes, what has not been seen is not just less likely, but effectively impossible.

Table 1 shows that regularization makes reconstruction possible even in the radically under-sampled regime where the number of parameters (here, 210, for n=20) exceeds the amount of data (here, 128 observations), and cross-validation leads to near-optimal results.

### 4.2. Re-Weighting Can Correct for Sampling Bias

To study bias correction, we simulate multiple examples of a biased sampling process. First, we construct landscapes (for a variety of β values) where answers to one of the questions are split, evenly, between YES (the “Type A” groups) and NO (the “Type B” groups). We then create two samples: a full sample of 256 observations, and a biased data sample, with 128 observations of Type-A groups, but only 64 observations of Type-B groups. This simulates an extreme form of bias, where the dominant Type-A cultures are over-sampled by a factor of 2:1.

We then compare the reconstruction performance in three conditions: the ideal case, with 256 observations; the naive-biased case, where parameters are learned from the biased sample; and the re-weighting case, where we implement the weighting prescription of Section 2.3.4. We measure both the KL divergence, and the average log-odds bias against the Type-B groups, defined as

(12) $$\mathrm{Bias}=\exp\left(\langle \log\frac{p_{B}}{p_{A}}\rangle \right)-1,$$

where $p_{B}$ is the model’s predicted probability of Type-B groups, $p_{A}$ (equal to $1-p_{B}$) is the predicted probability of Type-A groups, and the average is taken over multiple simulations in a β range. The true value, by construction, is $p_{A}$ equal to $p_{B}$, and negative values indicate bias against the minority cultures.

Table 2 shows the results; even at 2:1 levels of bias, our methods can achieve high reconstruction accuracy without inappropriately biasing the underlying landscape in favor of dominant cultures.

### 4.3. Partially Observed Data Can Be Consistently Integrated into Inference

**Figure 2:**
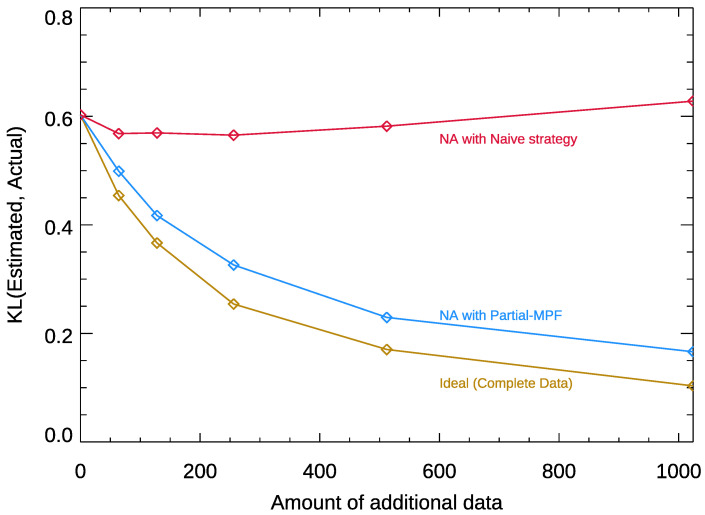
An example of how Partial-MPF adapts the baseline MPF algorithm to make use of partial data. We begin with 128 complete samples of a particular 20-question landscape (drawn from a distribution with β equal to 0.2), and then add additional, incomplete samples where five of the 20 questions are marked unknown. As more, but incomplete, data is added, the Partial-MPF fit (blue line) continues to improve, though not as fast as when the additional data is complete (yellow line). By contrast, the naive strategy (red line) often makes the fit worse, because imputation destroys implicit correlations.

To test the performance of Partial-MPF, we consider a scenario where we have a certain amount of complete data, and then add in new partially observed data. Figure 2 shows an example of how this works in practice for a single simulated system. We begin with 128 data points, and then add increasing amounts of data which is 25% incomplete (a random selection of five of the 20 features are blanked out.) We compare our method to a common “naive” choice of taking missing variables to have the most commonly observed value in the remainder.

The three lines show how fit quality changes as (1) more fully observed data is added (the ideal case); (2) partially observed data is added, and integrated in using the Partial-MPF strategy; and (3) partially observed data is added, using the naive strategy. While Partial-MPF is able to make good use of the data to improve the fit (the KL from the estimated landscape to the actual landscape declines), additional (noisy) data very often harms the quality of the naive fit beyond a certain point. Table 3 shows the average results in different regimes; the same pattern is observed.

## 5. Results: The Database of Religious History

We present our empirical results in four parts. First, in Section 5.1, we look at the values of the inferred parameters. The parameters suggest how we should read the underlying “logic” of the landscape: the key interactions that, in combination, make some configurations more consistent with constraints than others.

We then look at the landscape of configurations, as implied by the parameters. In Section 5.2, we show how it can be used to inform hypotheses in cases where data is inconsistent or missing; we take, as an example, the case of a cult in the ancient Mediterranean.

In Section 5.3, we show how to visualize the large-scale structure of the landscape—the topography of “peaks” (concentrated regions where religions tend to cluster), “valleys” (where underlying constraints make traditions harder to sustain), and “floodplains” (areas of configuration space where constraints are weaker, favoring diversity and variation). Finally, in Section 5.4, we show how to analyze the local neighbourhood of a configuration, which gives us a new window into the question of cultural evolution over time.

### 5.1. Parameter Interpretation and Landscape Logic

**Figure 3:**
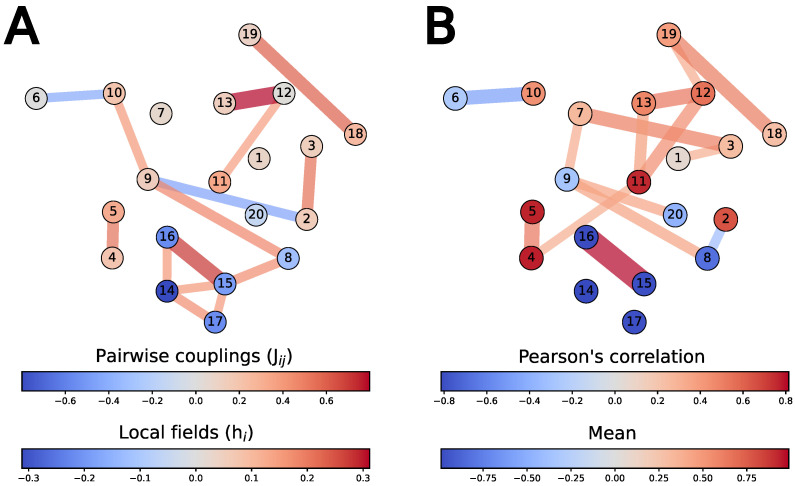
The logic of the cultural landscape (**A**), compared to the surface-level correlations (**B**). Nodes represent questions; see Table A1 for the question text. (**A**) edges represent the fifteen strongest pairwise couplings ($J_{ij}$) between questions, as inferred by Partial-MPF; nodes (questions) are colored by the value of the local fields $h_{i}$. (**B**) edges represent the fifteen strongest Pearson correlations; nodes are colored by the observed mean. Node placement (layout) is explained in the Appendix A.1.

Figure 3 provides a simple overview of the logic of the cultural landscape derived from the DRH. This compares the underlying parameters of the Inverse Ising model (the $J_{ij}$ and $h_{i}$ terms), inferred by Partial-MPF, to the surface-level, observed correlations in the data.

In some cases, the surface-level correlations are a good guide to the underlying logic. Our model suggests that, for example, the observed correlation between small-scale (18) and large-scale (19) rituals is most naturally explained, at this resolution, by an underlying sympathetic (i.e., $J_{18,19}$ positive) pairwise constraint. Similarly, the “big Gods” [33] pairing of supernatural monitoring (12) and supernatural punishment (13) is both a strong surface-level feature, and a core part of the landscape logic.

Much of the surface-level structure that we observe, however, turns out to be an emergent property of more complex relationships in the underlying parameters. The model suggests, for example, that a strong surface-level correlation between monumental architecture (3) and special treatment for corpses (7) can be explained away by mediation through other variables. Grave goods (9) is another example: it is rare in the observed data, but the local field for this feature is slightly positive, suggesting that there is nothing inherently difficult about maintaining a grave-good tradition. Instead, the practice is disfavored because of how it interacts with, for example, the keeping of written scriptures (2). Our model also reveals an underlying logic that links interactions among an “extreme” set of practices (castration (14), adult sacrifice (15), child sacrifice (16), grave-co-sacrifices (8), and suicide (17)).

### 5.2. Hypothesising the Unknown

Landscape models enable us to predict unknown data: given partial information about a group, Equation (1) allows us to conjecture about how the constraints, inferred from other systems, would interact in the particular case at hand. Cases with genuine expert disagreement, and cases where features of religious cultures are unknown due to the ravages of time, are the most exciting to analyze in this way.

As a particularly compelling example, consider the “Archaic Spartan Cults” (800 BCE—500 BCE). For these precursors to the Spartan state, both the presence of child sacrifice and small-scale rituals have been coded by the DRH expert as “unknown to the field”. In Table 4, we use the inferred parameters, along with what *is* known about the Spartans, to compute the degrees of belief in the four combinations.

The model is nearly 99% certain that the Cults did not practice child sacrifice. In this case, the known absence of both castration and adult sacrifice, both of which have sympathetic links with child sacrifice in the underlying model, are sources of evidence against the proposition (see Figure 3A).

The model is also reasonably confident about the presence of small-scale rituals; here, emergent constraints such as the strong pairwise coupling to the presence of large-scale rituals, which the Spartan Cults are known to have had, tilt the balance in favor of small-scale ritual. The judgement is less certain, however. The power of the Inverse Ising model is seen here not just in its recognition of common patterns, but in how it parses out the evidentiary value of different pieces of information.

### 5.3. The Landscape of Religious Culture

The basic output of our model is a probability distribution over $2^{20}$ possible configurations: a cultural landscape with peaks (small groups of high-probability configurations), and valleys (areas of low-probability configurations). As we shall see, landscapes can also include wider “floodplains”—more widely dispersed collections of configurations that are reasonably, and relatively equally, probable.

It is difficult, however, to visualize all the configurations at the same time: placing *all* the points of a 20-dimensional hypercube on a two-dimensional plot makes it hard to see which configurations are close (and, e.g., part of a connected plateau) vs. far (e.g., two well-separated peaks).

**Figure 4:**
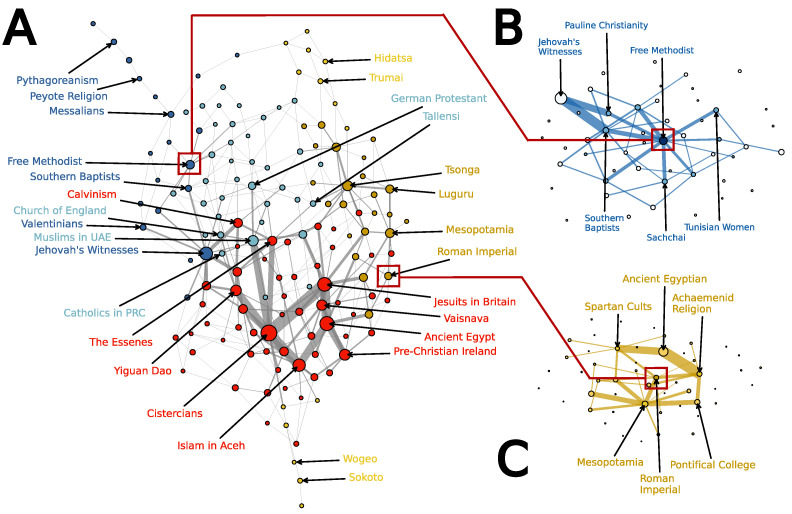
(**A**) (left) shows the 150 configurations that have the highest probability mass according to our model. We only show edges between configurations (nodes) that are immediate neighbors (separated by 1 Hamming distance). Nodes are scaled by the probability mass assigned to each configuration, and edges are scaled by the product of the probability mass of the nodes that they connect. Colors are assigned to each of five groups based on hierarchical clustering (see Appendix A.2 and the dendrogram, Figure A1). (**B**) shows the 50 most probable configurations in the local neighborhood of the Free Methodist Church, while (**C**) shows the 50 most probable configurations in the local neighborhood of the Roman Imperial Cult. In all cases (**A**–**C**), the layout is determined by a force-directed placement algorithm [34] as implemented in Graphviz [35]. For more on the layout approach, see Appendix A.1.

One way to approach this problem is to start with the topography of the most likely configurations. In Figure 4, we represent the 150 most probable configurations as a network, where configurations that differ in only one answer are connected by an edge, and the nodes are arranged to best represent distances; roughly speaking, configurations that differ in more answers are further apart (see Appendix A.1 for details). The configurations shown in the network represent 42% of the total probability mass, and provide an overview of the region of the landscape that contains the most favored configurations. Since we only visualize the 150 most probable configurations, a great deal of the landscape structure is not represented, including rarely explored parts of the space (e.g., configurations that support extreme practices, such as human sacrifice, suicide, and castration).

**Figure A1:**
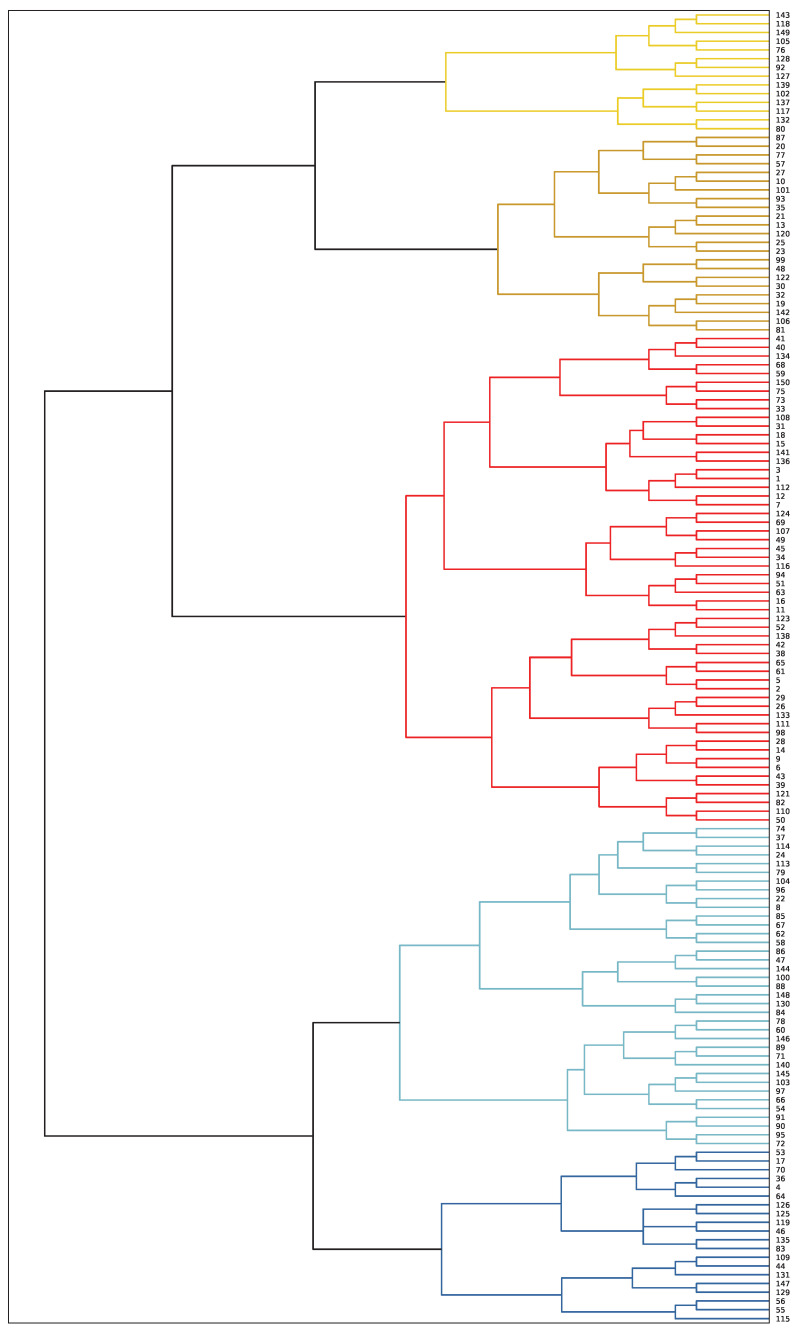
Dendrogram obtained by running an agglomerative clustering algorithm [43] on the 150 most probable configurations in our landscape. Labels correspond to the “Node” column in Table A3.

As a second aid to visualization, we used hierarchical clustering to construct a dendrogram (see Appendix Figure A1). Based on this clustering, we can split nodes into nested communities (see Appendix A.2). Table 5 provides names for the religions labeled in Figure 4A, and Table 6 (and Appendix B Table A3) provides a list of the most distinctive features of each group. The full list of groups is provided in Appendix B Table A3.

Group 1 (red) is the largest by probability mass (21%); it is characterized by a relative presence of small- and large-scale rituals, monuments, and scriptures. Among others, this group contains Ancient Egyptian religions, many Islamic traditions, and Catholic groups such as the Jesuits and Cistercians.

Group 2 (blue) is the second-largest (12%); it is characterized by a relative absence of small- and large-scale rituals, grave goods, and special corpse treatment. It includes demotic, charismatic, and reform traditions, including many Protestant groups such as the Southern Baptists, Jehovah’s Witnesses, and Pentecostalism. Group 2, in contrast to Group 1, is more evenly weighted among its configurations; where Group 1 has a small number of peaks, Group 2 is more like a “floodplain”. The configurations in the light blue group (2.1) tend to be found closer to Group 1 topologically; they have higher rates of state-political support than the configurations in the dark-blue group (2.2), and higher rates of both monuments and special treatment of corpses.

Finally, Group 3 (yellow) is the smallest by probability mass (9%). It is characterized by a relative absence of written scripture, and a relative presence of grave goods, and co-sacrifices in tombs. It includes many folk, traditional, indigenous, and “pre-Axial” [36] pagan cultures, such as the Iban traditional religion, Roman Imperial Cults and Mesopotamian religions. The light-yellow group (3.1), topologically further away from both Group 1 and Group 2, is characterized by a relative absence of moralizing “big Gods” [37] who conduct supernatural monitoring and punishment.

While the landscape is inferred without reference to time, cultural evolution appears to have explored the landscape in a somewhat sequential fashion. These temporal effects include shifts from Group 3’s pre-Axial tribal and archaic religious cultures towards Group 1’s later Axial religious cultures [36] and “big Gods” religions that co-evolved with large-scale complex societies [31,33]. Group-3 religions tend to be older than those in nearby Group 1, which has the highest concentration of religious cultures committed to a belief in high Gods. Group 2, in turn, includes popular developments out of Group-1 traditions into contemporary society, including many Protestant religions and more recent groups such as Pentecostalism, a sect established in the twentieth century and rapidly becoming one of the largest Christian sub-groups [33].

The landscape reflects more than just a temporal sequence of social, economic, and material evolution, however. It also seems to capture the constraints of more permanent features of the human mind. While Group 1 includes many later “solutions” to the constraints found by Axial-age and “Big Five” religions, it also includes cases such as pre-Christian Ireland. Religions, in other words, may co-evolve with social context, but they also have to respect the psychological constraints on how we believe and keep faith, and may well wander back to earlier solutions [38].

### 5.4. Focal Landscapes

Figure 4A provides an overview of how constraints combine to imply a landscape of configurations; a second possibility is to map the local landscape of configurations around a particular group. Among other things, this provides a grounded way to speculate on how a culture might evolve into the future, or where it might have come from—to ask, for example, which bits in a configuration might flip, and whether or not this would push the religion to a more probable configuration which is better able to satisfy the underlying constraints.

Figure 4B,C does this for two groups in our data, the (contemporary) Free Methodist Church and the (ancient) Roman Imperial Cult. In both cases, we show fifty nodes: the group itself as the focal node, and then the 49 most probable nearby configurations, which differ in up to two answers from the focal case.

As seen in Figure 4B, the Free Methodist Church is situated at a local peak, and all neighboring configurations are of lower probability. Some of them appear in our data (e.g., the Southern Baptists, and Pauline Christianity), but several are unoccupied. The highest probability configuration in the local region is occupied by the Jehovah’s Witnesses, two steps away.

The Free Methodist Church does not require participation in large-scale rituals. A change in this attribute is their most probable reformation (15%) and would place them in the same configuration as the Southern Baptists. This change would take the Free Methodist Church configuration closer to the local maximum, which is occupied by the Jehovah’s Witnesses. Another path to the Jehovah’s Witnesses configuration is through Pauline Christianity. However, all paths from the Free Methodist Church to the Jehovah’s Witnesses require intermediary states of lower probability. Slightly less probable is a mutation in which the Free Methodists adopt a practice for special treatment of corpses (13%). This reformation would take the Free Methodists in another direction in the landscape, and there is no religion in our dataset that corresponds to this configuration.

In contrast to the Free Methodist Church, the Roman Imperial Cult (Figure 4C) sits in a valley, with several neighboring configurations of higher probability. The Cult satisfies the constraints better without its own distinct written language, than with (as was actually the case), and with scriptures rather than without. Loss of its own distinct language would shift it up to the Mesopotamia configuration, while acquiring scriptures would shift it up to the Achaemenid configuration.

## 6. Discussion

The main goal of this work was to provide those in cultural evolution and sociophysics with new methods, and accompanying code, for inferring the landscapes beneath the incomplete data of the historical record. In addition to characterizing these methods through simulation, we showed how they play out in a real-world example, drawn from the Database of Religious History. In the words of archaeologist David Hurst Thomas, “it’s not what you find, it’s what you find out”, and we endeavored to show how landscape models not only organize data from the field, but provide insight into the underlying laws and dynamics that can help explain it.

A key direction for future research is to consider how these methods might be extended to even larger configuration spaces. As the number of features considered increase, so do the challenges; when *n* goes from 20 to 100, for example, the number of parameters goes from 210 to more than 5000. To maintain the same level of accuracy would, generically, require the amount of data to rise by a similar factor—however, this may not always be possible; in the final analysis, there are only a finite number of civilizations in human history.

A more creative solution to the problem is to go from the “unrestricted” Boltzmann machine case, where all $J_{ij}$s are (potentially) non-zero, to the “restricted” case, where some links are set to zero by the researcher ahead of time. In this case, the researcher sculpts a theory of constraints, restricting *a priori* the ways in which features may interact and reducing the number of free parameters. Another solution is to connect nodes not to each other, but to a small number of hidden variables—“layers”, in the deep-learning jargon. If there are *n* features, and *m* hidden nodes, then the total number of parameters, including local fields, is n(m+1), which may make the problem tractable again. Hidden layers have proven to be particularly expressive; in the physics jargon, they are equivalent to how renormalization leads to higher order interactions [39]. The original MPF paper [5] demonstrated the use of hidden nodes in this fashion, and the framework makes it possible to extend our Partial-MPF algorithm to these cases as well.

These are the are challenges in inference. There are equally compelling challenges in data curation itself. The DRH is one example of the exciting resources coming online for researchers in the human sciences, but these sources bring complexities of interpretation in their wake. As discussed in Appendix A.3, for example, drawing the boundaries between one group and another—in space, or time—is not a simple matter. This raises questions about how to properly combine the rich, qualitative data that comes from the field in ways that properly represent the diversity of human possibilities.

We paid particular attention to mitigating different forms of bias: both the bias that comes from undersampling a subset of traditions, and from how we treat missing data. There are other forms of bias in the data curation stage, however, and one we have not addressed is “question bias”: the ways in which the questions we use map the neighbourhoods of some cultures better than others.

One might imagine, for example, a set of questions very finely tuned to distinctions between different forms of Christianity, but that end up lumping indigenous practices in Africa into a single configuration. A scholar of Christianity might not, for example, include questions about whether a religious group has practitioners who are separately distinguished as “sorcerers” or “witches”, because the answer for all the traditions they have in mind would be “no”; the same question, however, could track important aspects of cultural evolution in other parts of the world (We thank one of our referees for this example). If we build a global landscape solely on the basis of “Christianity” questions, we will radically underestimate the diversity of indigenous traditions, and learn little about the network of constraints the stabilize these traditions.

A natural test for question bias is to check the extent to which “truly different” groups are mapped to the same configuration. If all of the groups in a particular region have an identical configuration, for example, or an usually low level of diversity, it might suggest that we are biased against important dimensions of the religious experience in that region.

Question bias is not, however, something that can be spotted or corrected purely at the algorithmic level. It may well be the case, for example, that one region truly has less religious diversity than another: the religions in a region may have emerged from a single founding group and undergone very little further evolution. Adding questions to artificially increase the diversity, in such cases, can do more harm than good—if the new questions are about somewhat accidental properties, they will increase noise without adding insight. In the final analysis, the proper construction of a landscape requires a proper choice of questions, which, in turn, requires sensitivity to the differences that matter.

## Figures and Tables

**Table 1:** Cross-validation can recover near-optimal sparsity parameters. Without sparsity, MPF consistently overfits to observed data. Reconstruction with 20 nodes (210 parameters), and 128 data points (i.e., the undersampled regime). The more computationally expensive $N_{2}$ strategy does not improve significantly over the simpler $N_{1}$.

β Range	Optimal KL	KL with CV	Standard MPF
	n **= 20, 128 Points**		
0.01–0.125 (dispersed)	0.22	0.23	1.2
0.125–0.25 (ordered)	0.55	0.56	2.3
0.25–0.5 (near critical)	0.62	0.63	19.4
0.5–1.0 (critical)	0.50	0.54	9.5

**Table 2:** Reweighting observations can correct for sample bias.

β Range	Ideal	Biased Sample			
		**KL**		**Bias against Minority**	
		**Corrected**	**Naive**	**Corrected**	**Naive**
0.01–0.125 (dispersed)	0.13	0.16	0.16	−0.2%	−14%
0.125–0.25 (ordered)	0.34	0.45	0.46	−0.1%	−40%
0.25–0.5 (near-critical)	0.43	0.55	0.60	9.6%	−51%
0.5–1.0 (critical)	0.48	0.57	0.71	0.1%	−65%

**Table 3:** Using Partial-MPF to reconstruct landscapes in the presence of partially-observed data. While the “naive” strategy actually decreases the quality of the fit, Partial-MPF enables efficient use of partial observations to improve knowledge of the landscape.

β Range	128 Full	128 Full + 128 Partial		256 Full
		**Partial-MPF**	**Naive**	
0.01–0.125 (dispersed)	0.23	0.17	0.23	0.15
0.125–0.25 (ordered)	0.56	0.41	0.56	0.34
0.25–0.5 (near critical)	0.63	0.44	0.81	0.40
0.5–1.0 (critical)	0.54	0.41	1.06	0.38

**Table 4:** Predictions of the landscape model for Archaic Spartan Cults.

	Small-Scale Ritual	No Small-Scale Ritual
Child sacrifice	1.2%	0.4%
No child sacrifice	**69.7%**	28.8%

**Table 5:** Observed configurations labelled in Figure 4A; DRH ID can be used as reference to the original source, e.g., DRH ID 654 (the Cistercians) can be found at https://religiondatabase.org/browse/654/#/.

Group	DRH ID	Entry Name (DRH)
Group 1	654	12th–13th c. Cistercians
	738	Ancient Egyptian
	931	The Society of Jesus (Jesuits) in Britain
	1043	Islam in Aceh
	852	Pre-Christian Religion / Paganism in Ireland
	1218	Yiguan Dao/I-Kuan Tao
	358	16th-17th c. Gaudiya Vaisnava Tradition
	456	The Essenes
	984	Calvinism (Early/Reformation)
Group 2	1311	Jehovah’s Witnesses
	855	Middle-Class Migrant Muslims in the UAE
	879	Free Methodist Church
	839	19th century German Protestantism
	1307	Southern Baptists
	906	The Church of England
	1392	Messalians
	859	Valentinians
	609	Tallensi
	1010	Pythagoreanism
	883	Catholics in the People’s Republic of China (PRC)
	1304	Peyote Religion (Peyotism)
Group 3	1251	Tsonga
	230	Religion in Mesopotamia
	1323	Luguru
	534	Roman Imperial Cult
	723	Trumai
	1511	Sokoto
	710	Hidatsa
	769	Wogeo

**Table 6:** Distinctive features of the five clusters in the landscape of Figure 4A; + indicates higher than average rates of “yes”; −, higher than average rates of “no”. See Appendix B Table A3 for full list.

Group	Color	Top Distinctive Practices
Group 1	Red	+ rituals (small, large); + monuments; + scriptures
Group 2	Blue	− rituals (small, large); − grave goods; − special corpse treatment
Group 3	Yellow	− scriptures; + grave goods; + co-sacrifices in tomb

## Data Availability

Data and open-source code (incl. optimized C code mpf_CMU) for the methods and analysis described in this paper is available at https://github.com/victor-m-p/cultural-landscapes, accessed on 24 January 2023.

## Appendix A. Database of Religious History: Analysis and Data Considerations

### Appendix A.1. Network Layout

As briefly touched upon in Section 5.3, it is mathematically impossible to faithfully represent the 20-dimensional hypercube landscape in a two-dimensional layout. By only laying out a subset of the possible configurations (e.g., in Figure 4A, 150 out of the total $2^{20}$ possible configurations), dimensionality reduction techniques can approximately compress the high-dimensional space into a low-dimensional spatial representation. We attempted approaches based on minimizing a global energy function (e.g. multi-dimensional scaling, and similar approaches [42]), as well as force-directed placement algorithms (e.g., Fruchterman–Reingold heuristic [34]). We achieved the most appealing results following the latter approach, using the algorithm as implemented in Graphviz [35]. We stuck with this approach for network layouts throughout, i.e., in all the plots shown in Figure 4, and in both plots shown in Figure 3. For the plots shown in Figure 4, the layout uses only immediate neighbors (1 Hamming distance) and is unweighted. To create the spatial layout of nodes for Figure 3, we thresholded the connections (couplings) between nodes (Figure 3A), such that only connections with an absolute coupling value above 0.15 are taken into account when running the force-directed algorithm. Figure 3B uses the layout obtained from Figure 3A to facilitate comparison.

### Appendix A.2. Hierarchical Clustering

We use agglomerative clustering as implemented in the Python package scikit-learn [43] to cluster nodes into nested groups. This produces the dendrogram shown in Figure A1. The algorithm starts off with all individual leaves (in our case configurations) in individual clusters and then successively merges nearby elements together. This results in a hierarchical grouping, where, e.g., the red clade is closer to the two yellow clades than it is to the two blue clades. There is no natural resolution of number of clades, as can be seen from Figure A1. Five clusters was chosen for clarity, but we emphasized the three overall splits between red, blue, and yellow, and we could equally well have used a higher resolution (i.e., more fine-grained clades). Notice that changing the resolution does not change the structure for the hierarchical clustering algorithm used here. This is in contrast to other common (non-hierarchical) approaches, such as Louvain community detection [44], which were found to give unstable results on our network of top configurations.

### Appendix A.3. Duplicate Entry Names

Entries in the Database of Religious History (DRH) all have a unique Entry ID, and the record has a name associated with it (Entry Name), but this name is not necessarily unique. In our sample of 407 religious cultures (Entry IDs), we have two religious cultures with the exact same Entry Name, Donatism and Roman Private Religion. In the case of Donatism, we do, in fact, appear to have two overlapping entries from different experts, one which focus on Donatism from 311 CE to 427 CE, and one which focus on Donatism from 311 CE to 600 CE. In this one case, a legitimate argument can be made that it would be more appropriate to collapse this into one record about Donatism, using the flexibility of our MPF algorithm to weight potential disagreement. The Roman Private Religion case is different, with both entries submitted by the same expert, one describing the religious culture between 202 BCE and 44 BCE, and the other one describing the religious culture between 600 BCE and 202 BCE. In this case, the periods do not overlap, and it is reasonable to assume that the expert has made the decision to split the records based on differences between the two cultures about which she has expert knowledge. These cases raise a more general point about independence, and how we should treat partially overlapping cultures.

The most extreme example from our curated subset of the DRH is the case of the Ancient Egyptian religions. We have a total of six different entries about Ancient Egyptian religions (e.g., early dynastic, first intermediate period, and old kingdom, etc.). Since these cultures naturally overlap on most attributes, the model will consider Ancient Egyptian religions as very stable configurations (which is, in fact, not totally unreasonable). Four of these six different entries focusing on Ancient Egyptian religions have the same configuration, and this configuration is assigned the second-highest probability mass in the landscape (annotated as “Ancient Egyption” in Figure 4A). Whether this is reasonable, or whether all of the Ancient Egyptian religions should be treated as one religious culture (and be weighted accordingly) is a difficult question. As culture is fluid, and no culture is completely independent from other—past and present—cultures, it seems impossible to design a general decision rule for whether to consider two cultures meaningfully independent. In this paper, we took the records from the DRH at face value, and treated each unique Entry ID as its own religious culture. In the future, more sophisticated approaches should be pursued in collaboration with domain experts.

## Appendix B. Database of Religious History: Religions and Question Codes used in This Analysis

**Table A1:** Question subset used in DRH analysis. “Long” question names, e.g., “Does the religion have official political support”, correspond to “Related Question” as they appear in the DRH, besides ending characters which have been modified, e.g., “Does the religious group have scriptures?” instead of “Does the religious group have scriptures:” as it appears in the DRH. Short question names are used for convenience. ID column was recoded to range from 1–20, and does not correspond to “Related Question ID” in the DRH.

ID	Question (Short; Long)
1	Official political support
	Does the religion have official political support
2	Scriptures
	Does the religious group have scriptures?
3	Monumental religious architecture
	Is monumental religious architecture present?
4	Spirit-body distinction
	Is a spirit-body distinction present?
5	Belief in afterlife
	Belief in afterlife?
6	Reincarnation in this world
	Reincarnation in this world?
7	Special treatment for corpses
	Are there special treatments for adherents’ corpses?
8	Co-sacrifices in tomb/burial
	Are co-sacrifices present in tomb/burial?
9	Grave goods
	Are grave goods present?
10	Formal burials
	Are formal burials present?
11	Supernatural beings present
	Are supernatural beings present?
12	Supernatural monitoring present
	Is supernatural monitoring present?
13	Supernatural beings punish
	Do supernatural beings mete out punishment?
14	Castration required
	Does membership in this religious group require castration?
15	Adult sacrifice required
	Does membership in this religious group require sacrifice of adults?
16	Child sacrifice required
	Does membership in this religious group require sacrifice of children?
17	Suicide required
	Does membership in this religious group require self-sacrifice (suicide)?
18	Small-scale rituals required
	Does membership in this religious group require participation in small-scale rituals (private, household)?
19	Large-scale rituals required
	Does membership in this religious group require participation in large-scale rituals?
20	Distinct written language
	Does the religious group in question possess its own distinct written language?

**Table A2:** For each community, we calculate the average possession of each of the 20 religious attributes. We weight this by the probability of each configuration in the community, and convert it to a percentage (Self). We compare this to the average possession of the attribute across all configurations that are not in the community. Again, we weight this by the probability of each configuration and convert it to a percentage (Other). We calculate the difference (S-O), and show the three attributes for which each community differ most from the rest.

Group	Color	Question	Self	Other	S-O
Group 1	Red	Small-scale rituals required	97.41	38.67	58.74
		Monumental religious architecture	79.64	40.41	39.23
		Scriptures	94.95	56.08	38.87
		Large-scale rituals required	100.00	65.77	34.23
		Reincarnation in this world	38.47	8.93	29.54
Group 2	Blue	Small-scale rituals required	22.25	86.72	−64.47
		Grave goods	7.43	57.19	−49.76
		Large-scale rituals required	50.90	95.99	−45.09
		Special treatment for corpses	44.53	87.74	−43.20
		Official political support	40.79	76.01	−35.22
Group 3	Yellow	Scriptures	19.37	90.21	−70.84
		Grave goods	86.97	30.62	56.35
		Co-sacrifices in tomb/burial	37.15	3.81	33.34
		Official political support	91.69	58.64	33.06
		Special treatment for corpses	98.27	68.76	29.51

Distinctive community features.

**Table A3:** For each observed religion, we count up the total probability mass which corresponds to configurations in either of the five communities. We assign the religion to the community which contains the configuration with the highest probability mass. Some observed religions will not appear in this table because none of their possible realized configurations correspond to data states in the top 150 most probable configurations. Some observed religions contain missing data or inconsistent coding. Complete records (religions that have a unique configurations) will appear with 100% weight, and incomplete records will appear for the community in which the sum is highest. DRH ID can be linked back to the original entry online; e.g., https://religiondatabase.org/browse/654/#/ links to DRH ID 654 (the Cistercians). Group 1 corresponds to the red community, Group 2.1 corresponds to the light-blue community, Group 2.2 corresponds to the dark-blue community, Group 3.1 corresponds to the light-yellow community, and Group 3.2 corresponds to the dark-yellow community (see Figure 4 and Figure A1).

Group	Node	DRH ID	Entry Name (DRH)	Weight
Group 1	1	654	12th–13th c Cistercians	100
Group 1	1	661	Congregation of Savigny	100
Group 1	1	899	Nahdlatul Ulama (NU)	100
Group 1	1	943	Moravian Missionaries in Nunatsiavut	100
Group 1	1	963	The Knights Templar	100
Group 1	1	966	Naqshbandī Order, Naqshbandī Tarīqa, Naqshbandīyyah, Khwājagān,	100
Group 1	1	974	Greek Chalcedonian Christians, Nicaea	100
Group 1	1	1293	The Zapotec or Ben ’Zaa (The Cloud People)	100
Group 1	1	676	The Order of the Holy Trinity for the Redemption of Captives, 1198–1500	55.01
Group 1	1	1466	Islamic modernists	51.06
Group 1	1	900	Pharisees	50.63
Group 1	1	965	Pachomian Monasticism	44.75
Group 1	1	888	Céli Dé monks	38.77
Group 1	1	843	Bnay Qyāmā and Bnāt Qyāmā	29.88
Group 1	1	691	Fur	28.28
Group 1	1	1268	Monotheistic Pre-Islamic South Arabia	26.53
Group 1	2	476	Cham Bani	100.00
Group 1	2	738	Ancient Egyptian	100
Group 1	2	788	The Late Bronze Age City-State of Ugarit	100
Group 1	2	970	Cult of Isis (Mysteries of Isis)	100
Group 1	2	1006	Ancient Egypt—Old Kingdom	100.00
Group 1	2	1008	Ancient Egypt—First Intermediate Period	100
Group 1	2	1125	Ancient Egypt—the Ramesside Period	100
Group 1	2	1069	Religion at Deir el-Medina	95.76
Group 1	2	1162	Lingbao dafa	93.52
Group 1	2	1258	Religion in Judah	93.52
Group 1	2	1191	Religion in Greco-Roman Alexandria	75.48
Group 1	2	228	Late Chosŏn Korea	53.15
Group 1	2	1149	Christianity in Tang China	35.99
Group 1	3	632	Local Religion at Selinous	100
Group 1	3	931	The Society of Jesus (Jesuits) in Britain	100
Group 1	3	1140	Early Medieval Confucianism	100
Group 1	3	354	Priests and Scholars of Hellenistic Uruk	98.01
Group 1	3	1004	Religion in Roman Ostia	51.50
Group 1	3	1227	Polytheistic Pre-Islamic South Arabia	32.14
Group 1	5	1043	Islam in Aceh	100.00
Group 1	5	1108	Early Christianity and Monasticism in Egypt	83.98
Group 1	6	852	Pre-Christian Religion/Paganism in Ireland	82.15
Group 1	6	1231	Shenxiao (“Divine Empyrean”) Daoism	54.45
Group 1	6	960	Bön (Bon)	52.93
Group 1	7	1218	Yiguan Dao/I-Kuan Tao	90.44
Group 1	9	358	16th-17th c. Gaudiya Vaisnava Tradition	73.72
Group 1	9	590	Huayan School (Early Tang)	51.70
Group 1	9	1172	Twofold Mystery (Chongxuan)	33.31
Group 1	11	914	Tariqa Shadhiliyya	100
Group 1	11	1370	Teda	69.30
Group 1	11	994	Monastic Communities of Lower Egypt: Nitria, Kellia, Scetis	35.27
Group 1	11	456	The Essenes	31.62
Group 1	12	416	Edinoverie	100.00
Group 1	12	984	Calvinism (Early/Reformation)	100
Group 1	15	442	Donatism	100.00
Group 1	15	483	Northern Irish Roman Catholics	100
Group 1	15	989	Opus Dei	100.00
Group 1	15	1038	Sino-Muslims in Qing China	100
Group 1	15	1321	Mourides (Muridiyya)	100
Group 1	15	387	Ahmadi; Ahmadiyya Muslim Jama’at; Ahmadiya	50.15
Group 1	15	927	Zealots	28.67
Group 1	18	176	Qumran Movement	62.40
Group 1	26	1295	Donatism	77.18
Group 1	26	621	Haitians	33.15
Group 1	28	869	Sichuan Esoteric Buddhist Cult	100
Group 1	29	891	Peruvian Mormons	100
Group 1	29	983	Tibetan and Himalayan Mundane and Landscape Cults	100
Group 1	29	493	Krishna Worship in North India—Modern Period	32.01
Group 1	29	441	Worshipers of Śītalā	29.47
Group 1	31	972	Nestorian Christianity	86.73
Group 1	31	1012	Inhabitants of Medieval Kurgus	83.58
Group 1	31	944	Mohyla’s Ukrainian Church	76.62
Group 1	33	200	Nechung Cult	100
Group 1	33	390	Dasara	100
Group 1	33	415	Shaiva World Renouncers	100
Group 1	33	440	Jain Digambara Tantra, Karnataka	100
Group 1	33	636	Balinese Śaiva priests (pedanda siwa)	68.74
Group 1	38	1041	Korean Catholicism	100
Group 1	39	850	Pre-Christian Religion / Paganism in Gaul	77.24
Group 1	41	420	Swaminarayan Sampraday	100
Group 1	41	227	Hindu Goddess Worship in Northwest India—Modern Period	83.93
Group 1	41	623	Diasporic American Hinduism	32.95
Group 1	42	1241	Taiping Movement	61.03
Group 1	42	870	Postsocialist Mongolian Buddhism	49.11
Group 1	42	934	Mongolian Buddhism during the Revolutionary Period	44.78
Group 1	43	1192	Kaharingan	100.00
Group 1	45	657	Trukese	23.23
Group 1	49	667	Ainu	58.28
Group 1	49	736	Jivaro	45.07
Group 1	49	1210	Mende	44.09
Group 1	49	717	Igbo	26.85
Group 1	49	1110	Twana	18.96
Group 1	59	563	Gaddi, a Hindu community of the Western Himalayas	100
Group 1	63	1178	Modern Mystery School (MMS)	100
Group 1	63	802	Chinese Nunnery in Myanmar	33.18
Group 1	69	886	Pushtimarg (The Path of Grace) in the UK and Gujarat	100
Group 1	75	714	Tamil Śaiva Bhakti	100
Group 1	75	926	Ladakhi Buddhism	100.00
Group 1	94	1247	Exovedate	100
Group 1	94	744	Rwala Bedouin	27.13
Group 1	108	526	Hmong Christianity	100.00
Group 1	108	564	Tribal Christianity (and allied castes) in the Himalayas	100
Group 1	110	686	Popoluca	53.45
Group 1	124	887	Postsocialist Mongolian Shamanism	84.17
Group 1	124	576	Kuy traditional religions	49.14
Group 1	133	958	Society of Jesus	100
Group 1	150	987	Parsis, Zoroastrians of India	100
Group 2.1	8	855	Middle-Class Migrant Muslims in the UAE	100
Group 2.1	8	1076	Inquisitors of Goa’s Santo Ofício	100
Group 2.1	22	1196	K’iche’ (Quiché)	68.03
Group 2.1	24	839	19th century German Protestantism	100
Group 2.1	24	1371	Twelver Shi’ism in post-revolutionary Iran	100
Group 2.1	37	1374	The Reformed Church (Early Orthodoxy)	100
Group 2.1	37	1522	Tijaniyya Order/	100
Group 2.1	37	906	The Church of England	62.60
Group 2.1	54	609	Tallensi	28.36
Group 2.1	58	977	Chishti Sufis	100
Group 2.1	58	883	Catholics in the People’s Republic of China (PRC)	99.63
Group 2.1	60	602	Amhara	26.91
Group 2.1	71	1517	Tunisian Women’s Associations	55.28
Group 2.1	79	1333	Cult of Thecla	90.43
Group 2.1	85	935	Nigerian Pentecostalism	100
Group 2.1	85	1127	Anglican Church of Korea	100
Group 2.1	85	1376	African Initiated Churches	53.25
Group 2.1	88	633	Mādhva	69.68
Group 2.1	95	884	Sub Saharan Africa Pentecostalism	100
Group 2.1	96	941	Chan Buddhists in early Qing period	86.26
Group 2.1	96	1024	Universal Salvation Ritual	34.38
Group 2.1	103	928	The Ghost Dance Movement and the Lakota Sioux	100
Group 2.1	104	1349	The Dingxiang Wang Cult	61.80
Group 2.1	130	419	The Worship of Jagannath in Puri (Odisha)	100
Group 2.1	145	607	Mohism	66.52
Group 2.1	146	637	Yahgan	90.63
Group 2.2	4	873	The Branch Davidians	100
Group 2.2	4	880	Egyptian Salafism (inluding North Africa and West Asia)	100
Group 2.2	4	968	Anabaptist Mennonites in North America, 1683–2021	100
Group 2.2	4	988	Churches of Christ- United States	100
Group 2.2	4	1311	Jehovah’s Witnesses	100
Group 2.2	4	858	The New Prophecy or “Montanism”	87.89
Group 2.2	4	897	Gaengjeongyudo	71.90
Group 2.2	4	948	Christianity in Ephesus	46.18
Group 2.2	4	196	Pauline Christianity (ca. 45–60 CE)	39.29
Group 2.2	4	1309	Circumcellions	31.63
Group 2.2	17	857	Wesleyanism	100
Group 2.2	17	879	Free Methodist Church	100.00
Group 2.2	17	942	African Methodist Episcopal Church	100
Group 2.2	17	950	The Religious Society of Friends	100
Group 2.2	17	975	Neo-Charismatic Movement—Third Wave Charismatic Movement	100.00
Group 2.2	17	1334	No-debt Movement in US Evangelicalism	100.00
Group 2.2	17	892	Charismatic Renewal Movement in Christianity—Second Wave Pentecostalism	96.88
Group 2.2	36	1307	Southern Baptists	100
Group 2.2	44	1392	Messalians	39.84
Group 2.2	46	859	Valentinians	83.50
Group 2.2	53	953	Sachchai	96.93
Group 2.2	55	1010	Pythagoreanism	66.18
Group 2.2	64	1013	Estado da Índia Renegades in Deccan	100
Group 2.2	64	182	Pauline Christianity	63.55
Group 2.2	109	1304	Peyote Religion (Peyotism) and the Native American Church	100
Group 2.2	109	812	Temple of the Jedi Order	81.19
Group 2.2	115	862	Ilm-e-Khshnoom	100.00
Group 2.2	125	842	American Evangelicalism	100.00
Group 2.2	125	915	Protestantism welcoming People with Disabilities	100.00
Group 2.2	129	871	Spiritualism	100
Group 3.1	76	768	Mundurucu	35.17
Group 3.1	76	723	Trumai	21.93
Group 3.1	80	1511	Sokoto	53.77
Group 3.1	92	710	Hidatsa	50.32
Group 3.1	105	689	Lengua	21.84
Group 3.1	127	677	Yapese	44.57
Group 3.1	128	658	Mapuche	87.90
Group 3.1	139	769	Wogeo	46.72
Group 3.1	143	764	Timbira (Canela)	95.67
Group 3.1	143	1228	Lesu	41.89
Group 3.1	149	1240	Azande	63.60
Group 3.1	149	737	Nama Hottentot	51.49
Group 3.1	149	1458	Mbuti	35.30
Group 3.2	10	1251	Tsonga	10
Group 3.2	10	794	Omaha	82.23
Group 3.2	10	389	Iban traditional religion	68.05
Group 3.2	10	492	Roman Divination	59.68
Group 3.2	10	748	Lamet	41.79
Group 3.2	10	729	Kikuyu	32.93
Group 3.2	10	1434	Kuna	28.65
Group 3.2	10	739	Lakalai	27.59
Group 3.2	13	230	Religion in Mesopotamia	100
Group 3.2	13	1129	Ancient Thessalians	100
Group 3.2	13	1337	Tell Afis (Syria)	55.01
Group 3.2	13	757	Kiribati	32.51
Group 3.2	13	1101	Natchez	22.65
Group 3.2	19	685	Badjau	82.79
Group 3.2	20	1323	Luguru	69.58
Group 3.2	21	993	Pontifex Maximus and Pontifices (Pontifical College)	100
Group 3.2	23	424	Achaemenid Religion	89.49
Group 3.2	23	681	Sargonic Empire	50.82
Group 3.2	25	534	Roman Imperial Cult	100
Group 3.2	25	1051	Religion at Tell el-Dab’a (ancient Avaris) in Ancient Egypt	98.74
Group 3.2	25	1189	Yādiya/Sam’al	90.74
Group 3.2	25	479	Mesopotamian city-state cults of the Early Dynastic periods	37.75
Group 3.2	27	1248	Religion in the Old Assyrian Period	100
Group 3.2	27	712	Huichol	27.33
Group 3.2	27	1015	Ancient Egypt—Predynastic Period—Early Naqada Culture	27.12
Group 3.2	32	721	Semang	89.41
Group 3.2	32	651	Kapauku	56.67
Group 3.2	35	470	Archaic Spartan Cults	69.65
Group 3.2	48	224	Old Norse Fornsed	42.32
Group 3.2	57	581	Mentawai (Rereiket)	39.64
Group 3.2	57	620	Papago	25.17
Group 3.2	77	662	Goajiro	51.79
Group 3.2	77	578	Gond	42.36
Group 3.2	81	650	Manus	61.11
Group 3.2	81	626	Barama River Carib	52.54
Group 3.2	81	614	!Kung	39.80
Group 3.2	87	1246	Shilluk	58.89
Group 3.2	87	733	Kaska	50.10
Group 3.2	99	722	Gros Ventre	92.31
Group 3.2	101	918	Fangshi	56.42
Group 3.2	120	257	Classic Zapotec	73.85
Group 3.2	120	671	Mbau Fijians	69.48
Group 3.2	120	1420	Creek	63.49
Group 3.2	120	732	Marquesans	19.45

**Table A4:** Religious cultures in our dataset that do not have configurations (or even possible configurations) which overlap with the 150 most probable configurations visualized in Figure 4.

DRH ID	Entry Name (DRH)
23	Late Shang Religion
173	Johannine Christianity
174	Matthew-James-Didache Movement
190	Sri Lankan Buddhism (1948-Present)
204	Han Confucianism
217	Roman private religion
222	Late Classic Lowland Maya
231	Roman private religion
263	Irish Catholicism
284	Yolngu religion
294	Xuanxue
308	Neo-Assyrian Scholars
310	Aztec Imperial Core
364	Chittagong Plain Buddhists
378	Bahinabai Chaudhari’s Songs: A Performance Tradition in Maharashtra
381	Sikhism: Guru Nanak to Guru Arjan
383	Varkaris
392	Sikhism: Guru Hargobind to Guru Gobind Singh
395	International Society for Krishna Consciousness (ISKCON)
400	Singaporean Mega-Churches
422	Demeter Cult
439	Śaiva Magic
455	Lan Na Buddhism
469	Won-Buddhism
472	Karma Kagyu or Kamtsang Kagyu
477	Tractarian Movement
478	Early Indian Buddhism
484	Northern Irish Protestants
485	Sámi pre-Christian religion
486	Church of Jesus Christ of Latter-day Saints (early)
490	Guglielmites
491	Anglican Church
520	Cham Ahiér
525	Church of Jesus Christ of Latter-day Saints (modern)
535	Pāśupatas
562	Medieval Srivaisnavism
570	Supreme Master Ching Hai World Society
580	Nāth Sampradāya
582	Siamese (Central Thai)
586	Kelantanese Thai Religion
589	Warrau
592	Veerashaivas
597	Confucianism - Eastern Zhou
599	Lepcha
605	Burmese
613	Maori
618	Nuxalk
619	Gilyak
624	The Roshaniyya
625	Copper Inuit
627	Tikopia
629	Newar Buddhists
630	Aranda
631	Cayapa
635	Ifugao
638	Klamath
639	Chinese Esoteric Buddhism (Tang Tantrism)
645	Meo Muslim, Mev, Mewati Muslim
646	Lakota Religious Traditions
647	Havasupai
649	Worshippers at the Chidambaram Nataraja Temple, Modern Period
652	Tiwi
655	Siriono
660	Iban
666	Siuai
669	Orokaiva
674	Aymara
675	Chukchee
679	Santal
680	Gujarati Mata Worshipers
682	Lakher
684	Huron
688	Darul Uloom Deoband
690	Ahl-e-Sunnat wa Jamaat
711	Kwoma
713	Northern Saulteaux
719	Hinduism in Trinidad
726	Early Sramanas
727	Raglai
742	Thai Bhikkhunis
745	Buka
749	Korean shamanism
751	Buddhism in the Mekong Delta
752	Haroi
755	Uyghur Islam
759	Comanche
765	The Oneida Community
770	Thai Forest Tradition
771	Muscular Christianity
826	The Church of Christ, Scientist
833	Dobu
841	Sa skya
846	Xuanzang’s Yogācāra Tradition
848	Sadducees
849	“Gaiwiio Religion,” “Longhouse Religion,” or “The Way of Handsome Lake” of the
	Seneca Tribe
851	Sikhism in the United States
854	pre-Christian Irish
860	Kimpa Vita
867	Nyingma Treasure
877	The International Network of Engaged Buddhists - INEB
882	Orphism
885	Contemporary West African Vodun
893	Sethian Gnostic
894	Universal Fellowship of Metropolitan Community Churches
896	Sannō Shintō
898	Rastafari of Jamaica
910	Christian Base Community movement
919	Catholicism in contemporary Croatia
921	Bhils
924	Muridiyya of Senegal
925	Rabbinic Judaism (Babylonia)
929	Drikung Kagyu
933	Marcionites
937	Vestal Virgins
938	Ugarit
939	Goodenough and Fergusson Islanders
940	Julio-Claudian Imperial Cult
946	Nyingma (rnying ma)
949	The Church of All Worlds
952	Ethiopian Jews
957	Spartan Religion
961	Romanian Orthodox Church
962	Amdo Gelukpa
964	Baul Fakirs of Bengal
967	Unitarian Universalism
969	Adi Dravida/Valluva Sakya Buddhism
971	Ganapatya
973	The Sarna religion of the Oraons of Jharkhand
976	Mising Community
978	Indonesian Catholicism
980	Pure Land Buddhist Schools in Early Medieval Japan
981	Nation of Islam
982	The Samaritans (Persian to early Roman periods)
985	The Victorines
986	Theurgy
991	Beat Buddhism
995	Tamil Neo-Saivism
997	Ancient Egypt - Early Dynastic Period
1028	German Pietists (Hasidei Ashkenaz)
1037	Third Intermediate Period in Ancient Egypt
1044	Han Imperial Cult under Emperor Wu
1060	The Taizhou Movement
1071	Digital Shinto Communities
1083	Drukpa Kagyü School (Bhutan)
1087	The Bogomils
1106	Old Kingdom Religion at Abydos
1109	Chan Buddhism in the Song
1133	Religion in Greco-Roman Egypt
1134	Tiantai
1136	The Fellowship of Goodness (Tongshanshe)
1153	Russian Orthodox Mission in Alaska
1154	The Cult of the Fox
1156	Solovetski monastery
1180	Batak Traditional Religions
1183	Religion at Nippur in the Ur III period
1197	Atheism in the Soviet Union
1199	Tiv
1223	Toda
1234	Ashanti
1279	Mandarese Muslims
1283	Butonese Muslims
1284	Religion of Phoenicia
1289	Buginese Muslims
1299	Russians (of Viriatino Village)
1300	Enlace de Agentes de Pastoral Indígena (EAPI, Network of Indigenous Ministry Agents)
1301	Moche (Mochica)
1312	Bishnoi
1319	Reginistas
1322	Pagans under the Emperor Julian
1335	Congregation of the Oratory
1341	Muslim Students Association of the United States and Canada
1344	Tibetan Nonsectarianism (ris med)
1352	Sakadvipiya Brahmanas
1357	Encratites
1386	Liumen (Liu School)
1390	Eastern Apache
1396	Ptolemaic Egypt—Egyptian Religion
1397	The Classic Period Peripheral Coastal Lowlands Ritual Ballgame Cult
1409	Formative Olmec
1412	Tenrikyo
1415	Anomeans
1419	Ancient Egyptian Religion in the Early 18th Dynasty
1426	Early Missionary Christianity in China
1433	Novatians
1436	The Monastic School of Gaza
1441	Mexica (Aztec) Religion
1454	Hesychastic Controversy
1468	Eastern Christianity From Nicaea to Chalcedon
1495	Secular Buddhists
1521	Order of the Hermits of St Augustine (Augustinian friars)
1542	Umbanda
